# Function of interleukin-17 and −35 in the blood of patients with hepatitis B-related liver cirrhosis

**DOI:** 10.3892/mmr.2014.2681

**Published:** 2014-10-16

**Authors:** MIN SHI, JUE WEI, JINBIN DONG, WENYING MENG, JIALI MA, TING WANG, NA WANG, YUGANG WANG

**Affiliations:** Department of Gastroenterology, Shanghai Changning Central Hospital, Shanghai 200336, P.R. China

**Keywords:** hepatitis B, liver cirrhosis, interleukin-35, interleukin-17

## Abstract

Intrahepatic T helper (Th)17 cytokine and serum interleukin (IL)-17 levels in patients with hepatitis B are positively correlated with the progression of liver cirrhosis (LC). IL-35 can significantly inhibit the differentiation of Th17 cells and the synthesis of IL-17. The present study aimed to investigate the function and expression of IL-17 and IL-35 in the blood of patients with hepatitis B-related LC. The levels of IL-17 and IL-35 in the peripheral blood of 30 patients with chronic hepatitis B (CHB), 79 with LC, 14 with chronic severe hepatitis B (CSHB), and 20 normal controls were detected by ELISA. Quantitative polymerase chain reaction was used to evaluate Epstein-Barr virus-induced gene 3 (EBI3), forkhead box (FOX)P3 and IL-17 mRNA expression levels in peripheral blood mononuclear cells (PBMCs). Western blotting was used to determine protein expression. The liver function of patients and normal controls was measured. EBI3, IL-17 and FOXP3 mRNA expression levels in PBMCs from patients with LC, CHB and CSHB were higher than those in cells from the controls. IL-17 mRNA levels differed significantly according to the Child-Pugh classification and exhibited an upward trend over time in contrast to a downward trend for EBI3 and FOXP3 mRNA. The changes in protein expression in the peripheral blood were consistent with the changes in mRNA expression. Serum IL-17 levels were positively correlated with total bilirubin (TBIL), alanine aminotransferase (ALT) and Child-Pugh grade, and were negatively correlated with albumin. These observed differences were significant. Serum IL-35 levels were negatively correlated with albumin, but not with Child-Pugh grade, ALT and TBIL. IL-17 and IL-35 may be critically involved in the pathogenesis of hepatitis B-related LC.

## Introduction

Hepatitis B virus (HBV) infection remains a global public health problem. More than 350 million people worldwide are suffering from persistent HBV infection (1). Chronic HBV infection is a serious clinical problem due to the potentially adverse sequelae, such as hepatic decompensation and the development of cirrhosis or hepatocellular carcinoma (2). The Child-Pugh classification is the common liver reserve function classification standard of cirrhosis, guides treatment and prognosis, and has very important reference value (3,4). The determination of Child-Pugh score, which may range from 5 to 15, is based on the presence and severity of ascites and hepatic encephalopathy, the prolongation of prothrombin time, and the levels of serum bilirubin and albumin. According to their Child-Pugh scores, patients are classified into three classes; A, B and C, with Child-Pugh scores 5–6, 7–9 and 10–15, respectively (5). The complication rate of chronic HBV is associated with the degree of viral replication, inflammation and fibrosis (6,7,8).

Cytokines are important chemical mediators synthesized and secreted from immune cells, which act on their corresponding receptors and regulate immune cell differentiation and proliferation, thus coordinating the immune and inflammatory responses and progression of fibrosis. In previous years, studies have demonstrated that intrahepatic T helper (Th)17 cytokines and serum interleukin (IL)-17 levels in patients with hepatitis B are positively correlated with the progression of liver cirrhosis (LC) (1,9). Cytokines do not act in isolation and are synthesized and secreted through mutual adjustment. They can regulate receptor expression and influence the biological effects of the cytokine network to achieve synergistic, antagonistic or functional expansion. IL-35 can significantly inhibit the differentiation of Th17 cells in addition to the synthesis of IL-17 (10). Thus far, few studies have investigated IL-17 and IL-35 in patients with LC. In the current study, the expression levels of IL-17 and −35 in the blood of patients with hepatitis B-related LC was investigated.

## Patients and methods

### Study objective

The present study included out- or inpatients treated at the Department of Gastroenterology and Infectious Diseases (Changning District Central Hospital, Shanghai, China), between December 2010 and June 2013. The patients included 30 with chronic hepatitis B (CHB), 79 with LC (27, 30 and 22 with Child-Pugh class A, B and C, respectively), 14 with chronic severe hepatitis B (CSHB), and 20 healthy controls. Peripheral blood was collected from each patient. All cases were diagnosed in accordance with the 2000 National Viral Hepatitis Prevention and Treatment Programs (11) and the 2005 Chronic Hepatitis B Prevention Guide (12). Patients with solid tumors, autoimmune diseases, other types of hepatitis virus infection or human immunodeficiency virus co-infection were excluded. The included patients had not received immunomodulatory and antiviral agents prior to treatment. The clinical characteristics of the patients are exhibited in Table I. The normal control (NC) group comprised 12 males and eight females, aged 22–64 years (mean, 32.5±9.0 years), from whom hepatitis B virus infection had been eliminated; exclusion criteria were the same as for the patients. The present study was approved by the Ethics Committee of Changning District Central Hospital, and all patients or their authorized family members gave signed informed consent.

### Materials and reagents

The FTC-3000 quantitative polymerase chain reaction (qPCR) instrument was obtained from Funglyn Biotech, Inc. (Scarborough, ON, Canada); the SYBR Green Real-Time PCR Master Mix was from Toyobo Co. (Osaka, Japan); primers for L-35 (Epstein-Barr virus-induced gene 3; EBI3), IL-17 and forkhead box P3 (FOXP3) were designed by the authors and synthesized by Generay Biotech Co., Ltd (Shanghai, China) (Table II); rabbit anti-IL-17 polyclonal antibodies were from Abcam (Cambridge, MA, USA); mouse anti-IL-35 monoclonal antibodies were from R&D Systems, Inc. (Minneapolis, MN, USA); rabbit anti-FOXP3 (D25D4) monoclonal antibodies were from Cell Signaling Technology, Inc. (Danvers, MA, USA); mouse anti-β-actin monoclonal antibodies, goat anti-rabbit immunoglobulin G (IgG) (whole molecule)-peroxidase antibodies and goat anti-mouse IgG (Fc specific)-peroxidase antibodies were from Sigma-Aldrich (St. Louis, MO, USA); the Bicinchoninic Acid (BCA) Protein Assay kit was from Thermo Fisher Scientific (Rockford, IL, USA); the Amersham ECL plus Western Blotting Detection System was from GE Healthcare Bio-Sciences (Pittsburgh, PA, USA); and the ELISA kit was from Uscn Life Science, Inc. (Wuhan, China).

### Isolation, cryopreservation and resuscitation of peripheral blood mononuclear cells (PBMCs)

Fresh blood (5 ml) was collected prior to surgery and at follow-up (1, 4 and 6 months), put into an EDTA anticoagulant tube, and sent to the laboratory within 2 h. The samples were centrifuged at 1,500 × g for 5 min to obtain the plasma, which was stored at −70°C. The remaining cell pellets were mixed with 3 ml phosphate-buffered saline (PBS) solution, added slowly to 3 ml prepared ficoll lymphocyte separation medium along the pipette wall and transferred to a horizontal rotor at 800 × g for 15 min. PBMCs in the intermediate layer were removed and added to a Falcon tube containing 3 ml PBS solution. The cells were washed at 200 × g for 10 min, added to 5 ml PBS solution following supernatant removal, washed once at 200 × g for 10 min, counted under a fluorescent microscope (Olympus IX51; Olympus, Tokyo, Japan) and then stored subsequent to supernatant removal. The cells in the three tubes were frozen with 500 μl RPMI-1640 medium containing 10% dimethyl sulfoxide and 10% fetal bovine serum, placed in a −80°C freezer overnight and transferred to liquid nitrogen for storage on the next day.

### qPCR

Total RNA was extracted by TRIzol^®^ reagent, and detected with ultraviolet spectrophotometry to determine RNA purity and concentration. Total RNA (2 μg) was reverse transcribed into cDNA. qPCR was performed with the ReverTra Ace qPCR RT kit (Toyobo Co.). Reverse transcription (RT) products were amplified for β-actin, IL-35 (EBI3), FOXP3 and IL-17 genes. The mRNA expression and gradation of each group were compared with a fully automated gel imaging analysis system (Gel Doc 2000; Bio-Rad, Hercules, CA, USA), subsequent to the amplified products undergoing gel electrophoresis. All results were corrected with reference to β-actin. The sequences and fragment lengths of the PCR primers are illustrated in Table II. qPCR was conducted under the following conditions: 40 cycles including initial denaturation at 94°C for 30 sec, denaturation at 94°C for 20 sec, annealing at 61°C for 30 sec, extension at 72°C for 30 sec and a final extension at 72°C for 1 min followed by termination at 4°C.

### Western blotting

PBMCs were added to sodium dodecyl sulfate (SDS) lysis solution (100–400 μl; mainly containing 50 mM Tris (pH 8.1), 1% SDS and inhibitors including sodium pyrophosphate, β-glycerophosphate, sodium orthovanadate, sodium fluoride, EDTA and leupeptin). Cells were subjected to high-speed homogenization with ice for 1 min, ultrasonic cracking three times within 20 sec, and centrifugation at 12,000 × g at 4°C for 20 min. The supernatant following centrifugation was transferred to a 0.5-ml centrifuge tube and stored at −80°C. The BCA protein assay method was used for the SDS-PAGE, transmembrane, immune response, development and gel image analysis of EBI3, FOXP3 and IL-17.

### Statistical analysis

SPSS version 13.0 (SPSS, Inc., Chicago, IL, USA) was used for statistical analysis and the values were expressed as the mean ± standard deviation. Numerical data were analyzed by the χ^2^ test, and quantitative differences between groups were processed by analysis of variance. Pair-wise comparisons were performed by the q test. The cumulative varicose vein rebleeding and survival rates were analyzed by a product-limit method (Kaplan-Meier) and all statistical tests were bilateral probability tests (α=0.05). P<0.05 was considered to indicate a statistically significant difference.

## Results

### Quantitative determination of EBI3, IL-17 and FOXP3 expression by qPCR

**Figure 1:**
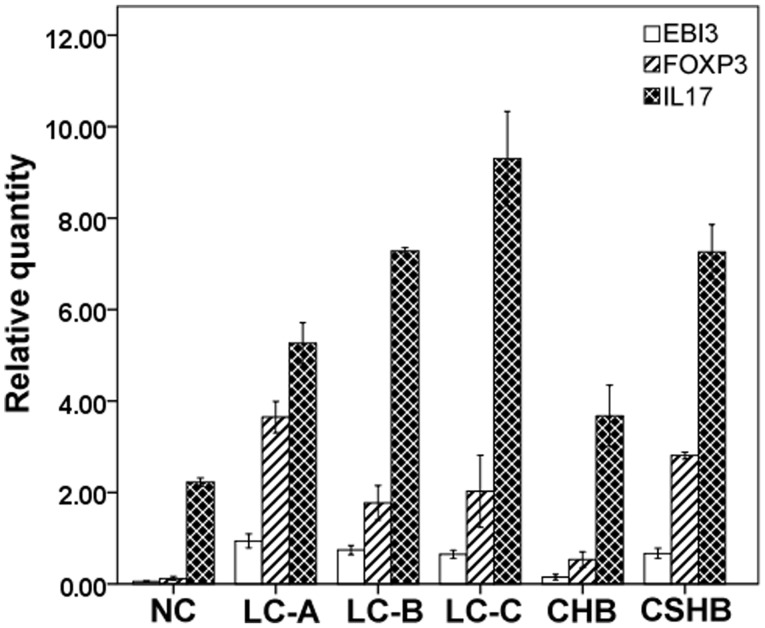
Fluorescent determination of EBI3, FOXP3 and IL-17 expression with polymerase chain reaction. NC, normal control; LC-A–C, liver cirrhosis Child-Pugh class A; LC-B, LC Child-Pugh class B; LC-C, LC Child-Pugh class C; CHB, chronic hepatitis B; CSHB, chronic severe hepatitis B, IL-17, interlaukin 17; EBI3, Epstein-Barr virus-induced gene 3; FOXP3, forkhead box P3.

The results of reverse transcription qPCR (RT-qPCR) are presented in Fig. 1. EBI3, IL-17 and FOXP3 mRNA expression levels in PBMCs from patients with LC, CHB and CSHB were significantly higher than those in the controls (P<0.01 or P<0.05). The levels of IL-17, EBI3 and FOXP3 mRNA differed significantly according to the Child-Pugh classification C or B vs. A. For EBI3: Child-Pugh A exhibited the highest expression; Child-Pugh C vs. A, P=0.002; Child-Pugh C vs*.* B, P=0.236; Child-Pugh B vs*.* A, P=0.019. For IL-17: Child-Pugh C exhibited the highest expression; Child-Pugh C vs*.* A, P<0.001; Child-Pugh C vs*.* B, P<0.001; Child-Pugh B vs*.* A, P<0.001. For FOXP3: Child-Pugh A exhibited the highest expression; Child-Pugh C vs*.* A, P<0.001; Child-Pugh C vs*.* B, P=0.373; Child-Pugh B vs*.* A, P<0.001.

Expression levels of EBI3, IL-17 and FOXP3 mRNA in patients with LC were significantly higher than in the CHB group (EBI3, Child-Pugh C/B/A vs. CHB, P<0.001 for all three comparisons; IL-17, Child-Pugh C/B/A vs. CHB, P<0.001 for all three comparisons; FOXP3, Child-Pugh C vs*.* CHB, P=0.001; Child-Pugh B vs*.* CHB, P=0.003; Child-Pugh A vs*.* CHB, P<0.001). There was also a significant difference in EBI3, IL-17 and FOXP3 mRNA expression between the different Child-Pugh classes compared with CSHB. For EBI3: Child-Pugh C vs*.* CSHB, P=0.820; Child-Pugh B vs*.* CSHB, P=0.330; Child-Pugh A vs*.* CSHB, P=0.003. For IL-17: Child-Pugh C vs*.* CSHB, P<0.001; Child-Pugh B vs*.* CSHB, P=0.966; Child-Pugh A vs*.* CSHB, P<0.001. For FOXP3: Child-Pugh C vs*.* CSHB, P=0.015; Child-Pugh B vs*.* CSHB, P=0.003; Child-Pugh A vs*.* CSHB, P=0.010.

### Determination of IL-35 (EBI3), FOXP3 and IL-17 expression levels with western blotting

**Figure 2:**
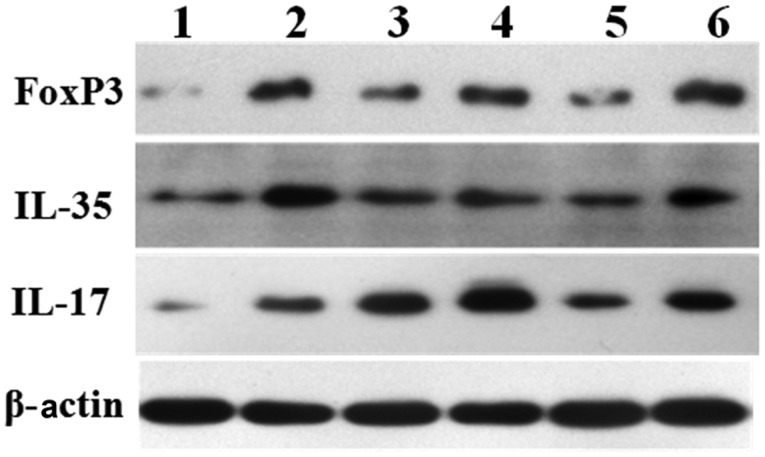
Changes in expression of IL-35 (Epstein-Barr virus-induced gene 3), IL-17 and FOXP3 by western blotting. Lanes: 1, normal control; 2, LC Child-Pugh class A; 3, LC Child-Pugh class B; 4, LC Child-Pugh class C; 5, chronic hepatitis B; 6, chronic severe hepatitis B. IL, interleukin; FOXP3, forkhead box P3; LC, liver cirrhosis.

Western blotting indicated that in patients with LC, CHB and CSHB, IL-35 (EBI3), FOXP3 and IL-17 protein expression levels were significantly higher than those in the NC group. Patients with LC with different Child-Pugh classes also demonstrated differences in EBI3, FOXP3 and IL-17 protein expression levels (EBI3, Child-Pugh A had relatively high expression; IL-17, Child-Pugh C had relatively high expression; and FOXP3, Child-Pugh A had relatively high expression). The change in protein expression was consistent with that observed in the mRNA expression (Fig. 2).

### Determination of IL-17 and IL-35 (EBI3) concentrations in the peripheral blood by ELISA

**Figure 3:**
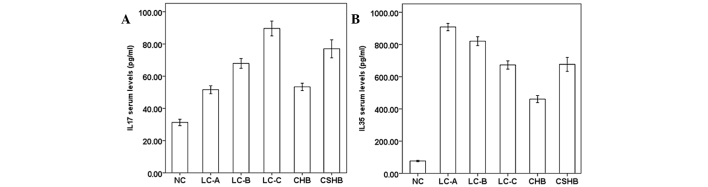
ELISA determination of IL-17 and IL-35 serum concentration. Serum concentrations of (A) IL-17 and (B) IL-35. IL, interleukin; NC, normal control; LC-A, liver cirrhosis Child-Pugh class A; LC-B, LC Child-Pugh class B; LC-C, LC Child-Pugh class C; CHB, chronic hepatitis B; CSHB, chronic severe hepatitis B.

The results of the ELISA demonstrated (Fig. 3) that, in LC, CHB and CSHB, the serum concentrations of IL-17 and IL-35 were significantly higher than those in the NC group (P<0.001). Patients with LC with different Child-Pugh classes demonstrated different EBI3 and IL-17 levels (EBI3, Child-Pugh A exhibited higher levels by pairwise comparison, P<0.001; IL-17, Child-Pugh C exhibited relatively high levels by pairwise comparison, P<0.001). In patients with LC Child-Pugh B and C, IL-17 and IL-35 concentrations in the peripheral blood were significantly higher than those in the CHB group (EBI3, P<0.001; IL-17, P<0.001). In patients in the LC group with Child-Pugh class A, IL-35 serum concentrations were higher than those in the CHB group (P<0.001), but IL-17 serum levels did not differ significantly (P=0.393). Fig. 3 demonstrates the differences in IL-35 and IL-17 serum levels between the LC and CSHB groups (IL-35, Child-Pugh C vs*.* CSHB, P=0.850; Child-Pugh B vs*.* CSHB, P<0.001; Child-Pugh A vs*.* CSHB, P<0.001; IL-17, Child-Pugh A–C vs*.* CSHB, P<0.001).

### Correlation analysis

Serum alanine aminotransferase (ALT), total bilirubin (TBIL) and albumin levels were compared with IL-17 and IL-35 levels. Correlation analysis identified that serum IL-17 and IL-35 levels were negatively correlated with albumin and serum IL-17 levels were significantly positively correlated with TBIL, ALT and Child-Pugh class (P<0.01). The serum IL-35 levels were negatively correlated with Child-Pugh class and demonstrated no correlation with ALT and TBIL (Table III).

## Discussion

IL-17 is predominantly produced by activated Th17 cells, and the serum IL-17 level reflects the number and function of Th17 cells to a certain extent (13–15). A study by Sun *et al* (16) suggested that the increase in Th17 cells in patients with LC promotes hepatic stellate cell activity, which leads to further disease progression. Ye *et al* (17) indicated that the number of IL-17-positive cells increases with stage of liver fibrosis. This may be associated with the crucial function that transforming growth factor (TGF)-β1 has in the development of liver fibrosis and the fact that cooperation between TGF-β1 and IL-6 may promote maturation of CD4^+^ T cells into Th17 cells (18–20). Th17 cells produce IL-6 and IL-17, which promote chronic liver inflammation, leading to the occurrence of cirrhosis. The current study identified that EBI3, IL-17 and FOXP3 mRNA and protein expression levels in PBMCs from patients with LC, CHB and CSHB were higher than those in the controls. Serum concentrations of IL-17 rose significantly, which were negatively correlated with albumin, but positively correlated with TBIL, ALT and Child-Pugh class. Serum IL-17 levels increased with LC Child-Pugh classification and were correlated with liver inflammation, necrosis and synthesis function, suggesting an important function in the occurrence and development of liver fibrosis.

IL-35 is a heterodimer that consists of the EBI3 protein and IL-12 p35 subunit (10,21,22). IL-35 is secreted mainly by T regulatory (Treg) cells. Its primary physiological function may be to induce the formation of Th1 cells and facilitate the proliferation of Treg cells (23). A previous study (10) established that IL-35 can inhibit differentiation of Th17 cells and IL-17 production and that IL-17 production is greater in spleen cells from EBI3-deficient mice. Liu *et al* (24) have demonstrated that EBI3 inhibits the responses of Th17, Th1, IL-2 and Treg. TGF-β can enhance the proinflammatory response by accelerating Th17 differentiation. IL-35 may upregulate interferon-γ, which can prevent phosphorylation of the TGF-β receptor downstream effector SMAD family member 3. This blocks TGF-β binding to its receptor and prevents the differentiation of Th17 cells (15). Retinoid-related orphan receptor γt (RORγt) is an important transcription factor of Th17 cell differentiation, and EBI3 deletion increased the expression of IL-17, IL-22 and RORγt (25). Several research groups have demonstrated that specific expression of FOXP3 in Treg cells is required for the Treg cell development and function (26–28).

The current study identified that patients with CHB, LC and CSHB, IL-17, IL-35 and FOXP3 mRNA and protein expression levels were higher than those in the NC group. Serum levels of IL-35 were significantly increased, which indicated that proinflammatory cytokines were increased in the cirrhotic process, while anti-inflammatory cytokine IL-35 levels were correspondingly upregulated. IL-17 mRNA and protein levels increased with LC Child-Pugh classification, whereas the expression of IL-35 mRNA and protein was reduced. The downregulated EBI3 content was also reduced following transcription into the EBI3 subunit and binding with the p35 subunit to form IL-35. Serum concentrations and protein expression levels are consistent with the mRNA. Further analysis demonstrated that serum IL-35 levels were negatively correlated with the LC Child-Pugh classification. The results indicate that in LC, reduced IL-35 expression hinders Treg cell amplification from secreting more IL-35. Thus, IL-35 cannot inhibit Th17 cell differentiation and IL-17 overexpression, excessive inflammation reactions are not prevented and the development of LC is promoted. The mRNA and protein levels of FOXP3 support this conclusion.

Previous studies have suggested that immune hyper-reactivity has an important function in the pathogenesis of severe hepatitis (29). It has been demonstrated that in patients with CHB, LC and CSHB, mRNA and protein levels of IL-17, IL-35 and FOXP3 are higher than those in the NC group, and the serum levels of IL-17 and IL-35 are significantly increased. Treg cells can suppress the immune response from over-reaction and reduce the immunopathological damage, however, they also inhibit the cytotoxic response against pathogens, which may lead to the exacerbation of infection.

The preliminary view is that IL-17 and IL-35 may be critically involved in the pathogenesis of hepatitis B-related LC. Studies on the association between IL-17 and IL-35 may further the understanding of the immune mechanisms involved in the progression of LC, and aid the development novel therapeutic targets. The present study had only a small number of cases and short follow-up; therefore, it was not possible to evaluate the dynamic changes in IL-17 and IL-35. Further studies with a larger number of samples and extended follow-up are warranted.

## Figures and Tables

**Table I:** Clinical characteristics of patients.

		Liver cirrhosis Child-Pugh class				
				
Clinical feature	NC	A	B	C	CHB	CSHB
Case (n)	20	27	30	22	30	14
Gender						
Male (n)	12	21	25	19	22	12
Female (n)	8	6	5	3	8	2
Age (years)^a^	39.5±9.0	45.4±7.2	52.9±8.9	56.9±9.8	38.1±6.7	47.0±8.2

a Data are presented as the mean ± standard deviation.

NC, normal control; CHB, chronic hepatitis B; CSHB, chronic severe hepatitis B.

**Table II:** Sequences for reverse transcription quantitative polymerase chain reaction.

mRNA	Sequence (5′-3′)	Amplicon size (bp)
IL-35 (EBI3)	F: TCATTGCCACGTACAGGCTC R: GGGTCGGGCTTGATGATGTG	208
IL-17	F: AGATTACTACAACCGATCCACCT R: GGGGACAGAGTTCATGTGGTA	151
Forkhead box P3	F: GTGGCCCGGATGTGAGAAG R: GGAGCCCTTGTCGGATGATG	238
β-actin	F: TGGAGAAAATCTGGCACCA R: CAGGCGTACAGGGATAGCAC	189

IL, interleukin; EBI3, Epstein-Barr virus-induced gene 3; F, forward; R, reverse.

**Table III:** Correlations between liver function and serum concentrations of IL-17 and IL-35.

	IL-17		IL-35	
		
	r	P	r	P
Total bilirubin	0.338	<0.001	0.122	0.146
Albumin	−0.698	<0.001	−0.384	<0.001
Alanine aminotransferase	0.248	0.003	0.038	0.651
Child-Pugh classification	0.873	<0.001	−0.798	<0.001

IL, interleukin.
